# Assessing the effect of therapeutic level of oxytetracycline dihydrate on pharmacokinetics and biosafety in *Oncorhynchus mykiss* (Walbaum, 1792)

**DOI:** 10.1038/s41598-024-73921-8

**Published:** 2024-10-01

**Authors:** Richa Pathak, Sumanta Kumar Mallik, Prasanna Kumar Patil, Krishna Kala, Neetu Shahi, Ranjit Kumar Nadella, Nityanand Pandey, Kishor Kunal, Pramod Kumar Pandey

**Affiliations:** 1 ICAR-Directorate of Coldwater Fisheries Research (ICAR-DCFR), Anusandhan Bhavan, Industrial Area, Bhimtal, Nainital, 263 136 Uttarakhand India; 2 ICAR- Central Institute of Brackishwater Aquaculture (ICAR-CIBA), Raja Annamalai Puram, Chennai, 600028 Tamil Nadu India; 3 ICAR- Central Institute of Fisheries Technology (ICAR-CIFT), CIFT Junction, Willingdon Island, Matsyapuri, Kochi, 682 029 India

**Keywords:** Oxytetracycline dihydrate, Rainbow trout, Drug absorption kinetics, Antibiotic residue, Non-compartmental analysis, Zoology, Environmental sciences

## Abstract

The aim of the experiment was to investigate the pharmacokinetics of oxytetracycline dihydrate after a single oral administration of 80 mg kg^−1^ day^−1^ in rainbow trout and assess its biosafety at concentration of 80, 240, 400, and 800 mg kg^−1^ day^−1^ over 30 days, focusing on various aspects such as effective feed consumption, physiological responses, drug tolerance, and detection of low drug concentrations in rainbow trout. The pharmacokinetics study spanned a duration of 5 days, while the assessment of biosafety extended for a 30-day safety margin, followed by a subsequent 10-day residual analysis. Pharmacokinetic analysis revealed slow absorption with low-rate constant in tissues. Absorption rates vary among tissues, with the gill showing the highest rate (0.011 h^−1^) and plasma exhibiting the slowest (0.0002 h^−1^). According to pharmacokinetic analysis, the highest concentration, C_max_ (µg kg^−1^) was observed in the kidney (9380 µg kg^−1^) and gill (8710 µg kg^−1^), and lowest in muscle (2460 µg kg^−1^). The time (T_max_) to reach peak concentration (C_max_) varied among tissues, ranging from 3 h in the gill to 32 h in the muscle, with 24 h in plasma, 32 h in the kidney, and 16 h in both the liver and skin. The liver and kidney had the highest area under the concentration-time curve (AUC_(0−128)_), indicating widespread drug distribution. Prolonged elimination occurred at varying rates across tissues, with the gill showing the highest rate. The study found that OTC concentrations exceeded the LOD and LOQ values. Biosafety evaluation showed effective feed consumption, physiological responses, and low drug concentrations in muscle at the recommended dosage of 80 mg kg^−1^ fish day^−1^.

## Introduction

Global fish production, encompassing crustaceans, molluscs, and various aquatic species, has exceeded 100 million tons, with aquaculture contributing 52% to human consumption since 2018^1^. It is regarded as a significant worldwide source of animal protein, supplying approximately 20.5 kg per person annaully^2^. In the realm of aquaculture, oxytetracycline dihydrate (OTC), a versatile antibiotic, plays a pivotal role in treating bacterial infections in farmed fish^3^. Due to its proven efficacy against various bacterial strains, this tetracycline-class antimicrobial agent is frequently employed for both treatment and prevention. It is commonly utilized in veterinary medicine due to its broad-spectrum activity, ability to permeate body fluids and tissues, and cost-effectiveness^4^. The prevalence of oxytetracycline extends to aquaculture, where it stands out as one of the most frequently used antimicrobial agents, demonstrating efficacy against both Gram-negative and Gram-positive bacteria^5^. Regulatory bodies, such as the United States Food and Drug Administration (USFDA) and the European Commission, have approved the use of oxytetracycline in aquaculture as a feed additive to combat bacterial diseases in fish^6,7^. But, the use of antibiotics to promote livestock growth not only in human and veterinary medicine but also in agriculture, has raised concerns about environmental pollution. The surge in aquaculture practices, particularly in developing countries lacking proper regulations, has led to indiscriminate antibiotic use for fish health management, posing the risk of development of antibiotic resistance in the culture environment. Various studies have pointed out the potential hepatotoxicity and nephrotoxicity of antibiotics in several fish species, causing damage to cell membranes, mitochondria, and respiration^8–12^.

Rainbow trout (*Oncorhynchus mykiss*), a freshwater fish species, holds significant global importance in aquaculture due to its economic value. However, the emergence of disease outbreaks and health challenges stemming from various microbial infections poses a substantial threat to trout farming. This has led to a surge in antibiotic usage for therapeutic and prophylactic purposes, often without a comprehensive understanding of pharmacokinetics, biosafety, and treatment efficacy under diverse culture conditions. The pharmacokinetics of oxytetracycline in various fish species have been studied, including rainbow trout (*O. mykiss*), channel catfish (*Lctalurus punctatus*), Atlantic salmon (*Salmo salar*), yellow perch (*Perca flavescens*), sea bass (*Dicentrarchus labrax*), grass carp (*Ctenopharyngodon idella*), and tilapia, *Oreochromis niloticus*^3,13–20^. Pharmacokinetic parameters directly influence the therapeutic outcomes of antibiotics. Although there is an abundance of pharmacokinetic data in fish, aiding in establishing optimal dosing regimens and minimizing environmental impacts, variations exist among fish species due to differences in the antibacterial properties of microbial agents. Furthermore, elevated doses are sometimes necessary to achieve therapeutic efficacy. Therefore, comprehensive studies on antibiotic pharmacokinetics across various economically important fish species in aquaculture are imperative, considering their specific culture conditions. Such studies would enhance our understanding of drug distribution, absorption, and elimination within fish bodies. In addition to that the rationality of study of the biosafety of oxytetracycline dihydrate in fish is crucial due to its potential deleterious effects on fish health. The recent studies have highlighted potential liver and kidney damage from antibiotics in fish, emphasizing the need for optimizing dosing to ensure survival of the fish in culture environment. Understanding the drug biosafety is also critical for establishing effective dosing, enhancing treatment efficacy, mitigating environmental risks, and ensuring responsible use in commercially significant fish species as the indiscriminate use of antibiotics may lead to residual issues in fish tissues and products, posing hazards to consumers^21^. Tetracycline and macrolides have been identified as genetically toxic to fish^22^. Thus, evaluating the biosafety of oxytetracycline is essential to comprehend the potential risks and consequences linked to its application in aquaculture. The current investigation aims to evaluate the impact of a single dose oral administration of oxytetracycline dihydrate on pharmacokinetics and its varied concentrations; 80, 240, 400, and 800 mg kg^−1^ fish day^−1^ on the biosafety of rainbow trout under a water temperature of 20 °C.

## Materials and methods

### Appropriate ethics declarations

The experimental techniques utilized and the involvement of rainbow trout, *Oncorhynchus mykiss* (Walbaum, 1792) in this study were approved by the Institute Animal Care and Use Committee (IACUC) (File no. ICAR-DCFR/IACUC/12/06/2020/07), ICAR-Directorate of Coldwater Fisheries Research, Bhimtal, India and also in accordance to the Ministry of Science and Technology, Government of India under the Rules for “Manufacture, Use/Import/Export and Storage of Hazardous Microorganisms/ Genetically Engineered Organisms or Cells, 1989 (Rules 1989) of Environment (Protection) Act 1986.” The study was performed following the relevant guidelines and regulations. All the methods and results stated in the study were in accordance with ARRIVE guidelines and regulations.

### Chemical

Oxytetracycline dihydrate (Cat. No. O4636-10G) was obtained from Sigma Aldrich, US. Merck provided ammonium hydroxide, ammonium acetate, ethyl acetate, and hexane (Darmstadt, Germany). J.T. Baker supplied the acetonitrile (ACN). Millipore’s Milli-Q technology was used to get purified water (Pal Scientific, USA) for use in the current pharmacokinetic and biosafety study.

### Experimental fish

Rainbow trout juveniles (average weight of 152.2 ± 24.1 g and an average length of 21.8 ± 0.21 cm) were procured from the system of re-circulatory aquaculture system (RAS), ICAR-DCFR, Bhimtal. These fish were then distributed into circular fiberglass reinforced plastic (FRP) tanks with a 1-ton capacity, with each tank containing 36 fish, and the experiment was conducted in triplicates with treatment and control groups. The fish were allowed to acclimatize for 15 days and were fed a drug-free diet at 2.0% of their body weight once a day before the trial to ensure better feed acceptance.

### The preparation of medicated feed

The medicated feed included a binder consisting of 5% vegetable oil. Oxytetracycline dihydrate was added to the vegetable oil at a concentration of 80 mg kg^−1^ fish body weight. The drug was thoroughly mixed with the feed, ensuring an even distribution based on the fish body weight. The quantity of the feed was calculated based on the 1% of fish biomass, in the experimental tank for mixing of antibiotic. Subsequently, the feed was dried for a period of 30 min in the absence of light and stored at a temperature of 4 °C for immediate use. In the control group, the same method was employed for feed preparation, but no antibiotic was incorporated.

### Physico-chemical parameters

The water quality parameters; pH, dissolved oxygen, alkalinity, water temperature, calcium hardness, ammonia, nitrite, and nitrate were measured daily in pharmacokinetic experiment, while in case of biosafety experiment the parameters were measured twice a week by conventional procedures for water and wastewater examination^23^.

### Administration and sampling

The fish were starved for 12 h before administration of the medicated feed. The rainbow trout were orally administered a single dose of oxytetracycline dihydrate at 80 mg kg^−1^ fish body weight through gavaging in semi liquid form. Various samples including plasma, liver, kidney, muscle, gill, and skin were collected at different time points; control (0, 24, 48, 64, 96 h) and treatment (0, 2, 4, 8, 12, 16, 24, 32, 48, 64, 96, 128 h) after the oral gavaging of the drug through feed. In each tank, three rainbow trout were collected, pooled, and processed to obtain the tissue samples as mentioned above. Blood samples were taken from the caudal vein using a heparinized 1 mL syringe and centrifuged immediately at 1000 xg for 10 min to obtain plasma. All samples were frozen and stored at -80 °C for later analysis of antibiotic residue by LC-MS/MS. The experimental trial was conducted at 20 °C.

### Sample preparation protocol for LC-MS/MS analysis of oxytetracycline (OTC)

Five grams (5 g) of homogenized tissue samples were accurately weighed and transferred into a 50 mL centrifuge tube. To the tube, 10 mL of 0.1% formic acid in acetonitrile, 10 mL of 0.05 M EDTA, and 5 mL of n-hexane were added. The mixture was thoroughly blended using a vortex for 1 min, followed by incubation in a shaker incubator at 650 rpm for 30 min. After incubation, the tube was centrifuged at 1800 xg for 10 min at 4 °C. The resulting supernatant was carefully transferred to a 15 mL centrifuge tube. The Oasis HLB column was conditioned with 1 mL of acetonitrile: water (1:1) and attached to 15 mL reservoirs equipped with frits at the bottom of the SPE cartridges. Slowly, hyperanized the sample was passed through the column, followed by a washing step with 0.5 mL of acetonitrile: water (1:1). A total of 2.5 mL of the eluent was collected. Before LC-MS/MS QTRAP analysis, the collected sample was filtered through a 0.2-micron RC filter into LC-MS vials.

### Instrumentation and analytical conditions

The analysis was performed using an AB SciexExion Liquid chromatograph equipped with a binary pump and an autosampler featuring a refrigerated sample tray. Separation of the analytes was achieved using a Saphire C18 3.5 μm; 150 × 4.6 mm (Waters) column. The autosampler tray temperature was maintained at 100 °C, while the column temperature was set to 40 °C. The mobile phase consisted of two components: Mobile phase A, which was composed of 0.1% formic acid in water (w/v), and mobile phase B, which contained 0.1% formic acid in methanol (w/v).

### Pharmacokinetics study

The pharmacokinetic parameters of oxytetracycline dihydrate were assessed using a non-compartmental pharmacokinetic model grounded in statistical moment theory. The calculation of the area under the concentration-time curve (AUC) employed the trapezoidal method, with the formula:

$$\:{\text{AUC}}\text{T}\text{r}\text{a}\text{p}\text{e}\text{z}\text{o}\text{i}\text{d}\text{}=21\text{}\sum\:i=1n-1\text{}(Ci\text{}+Ci+1\text{})\cdot\:(ti+1\text{}-ti\text{})$$

where *n* is the number of observed time points, $\:Ci$ and $\:Ci+1$ are the concentrations at time points $\:ti$ and $\:ti+1$ respectively, $\:ti$ and $\:ti+1$are consecutive time points

The absorption rate constant (*K*_*a*_) calculated as.

$${K_a}={\text{ }}ln{\text{ }}\left( 2 \right)/{t_{1/2a}}$$

$$\:{t}_{1/2}=\frac{\text{l}\text{n}\left(2\right)}{k}\text{}$$

where, $\:k$ is the elimination rate constant.

The linearity of the calibration curves was utilized to determine the limits of detection (LOD) and quantification (LOQ) by using the slope and the standard deviation (σ) of the intercept of the analytical curve. LOD was calculated as 3 × (standard deviation of the intercept/slope), and LOQ as 10 × (standard deviation of the intercept/slope). Recovery experiments were conducted by spiking the blank matrix with known standards, and the percent recovery was calculated using the formula: Recovery = (observed concentration of the spiked sample / expected concentration) × 100. The *C*_*max*_ and *T*_*max*_ were directly taken from analytical data.

### Biosafety of oxytetracycline

#### Experimental design

Rainbow trout juveniles with an average length of 16.8 ± 0.11 cm and a weight of 110.2 ± 15.1 g underwent a twenty-day acclimatization in FRP tanks at the ICAR-DCFR Wet laboratory. During this time, they received standard feed twice daily. The experiment comprised five groups: one acted as the control, whereas the remaining four served as the experimental groups, and each experimental group contained 30 rainbow trout juveniles. The groups were categorized based on daily consumption of graded concentrations of oxytetracycline dihydrate; 1 × (80 mg kg^−1^ fish day^−1^), 3 × (240 mg kg^−1^ fish day^−1^), 5 × (400 mg kg^−1^ fish day^−1^), and 10 × (800 mg kg^−1^ fish day^−1^). The rainbow trout juvenile ingested the medicated feed with the desired antibiotic concentration shortly after broadcasting. The fish were provided with an antibiotic-free feed for 10 days after 30 days of antibiotic therapy. The control group of fish received pelleted feed without antibiotics throughout the trial.

#### Preparation of medicated feed

In the formulation of the medicated feed, vegetable oil was added as a binder Designated concentrations of oxytetracycline dihydrate, based on fish body weight, were blended with 5% vegetable oil. The feed corresponding to the fish body weight was incorporated into this mixture and stirred until an even coating of the mixture (vegetable oil and antibiotics) was achieved. Subsequently, the feed was dried in the dark for 1 h and then stored at 4 °C for future use^24^. A similar process was employed to prepare the control feed, excluding the addition of antibiotics.

### Collection of samples

During the acclimatization phase, the antibiotic feeding period, and once every five days during the post-antibiotic feeding period, the animal behavior, percentage of feed consumed, and survival percentage of the control and treatment groups were examined daily. Three experimental fish from each tank were randomly collected on the 10th, 20th, 30th, and 40th day of the trial. Muscle, liver, kidney, and intestine were collected and preserved at -80 °C. Muscle tissues collected were subjected to analysis of the antibiotic residues.

### Hematological and biochemical analysis of blood

Hematological parameters were evaluated by drawing blood from the caudal vein using a 1 mL syringe and collecting it into an EDTA-coated tube. Red and white blood cells were diluted using specific solutions and counted under a microscope with Neubauer’s hemocytometer. Hemoglobin levels were measured using Sahli’s hemoglobinometer^25^. A commercial kit was employed for the analysis of plasma. The biuret technique was utilized to determine the total protein content^26^, while the BCG dye method was employed for the assessment of albumin content^27^. Blood glucose levels were determined using the glucose oxidase technique^28^. Globulin levels were calculated by subtracting the albumin content from the total protein.

### Plasma immunomodulation

A kinetic technique, facilitated by a commercial kit, was employed to quantify the creatinine levels^29^. The activities of aspartate transaminase (AST) and alanine aminotransferase (ALT) enzymes were assessed using the International Federation for Clinical Chemistry (IFCC) technique, while the activity of alkaline phosphatase (ALP) was determined using a method previously published^30^.

### Histopathological examination

The standard method was followed for the histological preparation. In brief, the different tissue samples of muscle, liver, kidney, and intestine collected from both treatment and control fish groups were fixed in Davidson fixative for 18 to 24 h before being replaced with 70% ethanol. The tissue blocks were sectioned using a microtome (4.0 μm, Microm HM 323, Thermo Scientific), followed by staining with hematoxylin and eosin^31^. The stained slides were mounted with DPX (Dibutylphthalate Polystyrene Xylene) and a microscope cover glass. Examination of histological changes was conducted using a light microscope (Olympus IX53, Camera Q-IMAGING, 01-MP 3.3-R-CLR-10, Color RTV10 BIT, Light source OLYMPUS, TH4-200).

### Validation and optimization of OTC using LC/MS/MS

According to EU Commission regulation No 37/2010 OTC MRL has fixed 100 µg kg^-1^ for finfish. Method validation was according to the guideline described in Commission Decision 2021/808/EC^32^. The withdrawal time estimation for OTC in muscle in biosafety were calculated using the WT 1.4 software, developed by Dr. P. Hekman and using the European Medicines Agency guidelines^33^.

### Statistical analysis

Data analysis was carried out using one-way analysis of variance (ANOVA) for pharmacokinetic and biosafety studies. The results were presented as mean ± SD (standard deviation of the mean) and deemed significant at *p* < 0.05. Differences between groups were assessed using Student’s t-test, specifically comparing treatments against the control group to determine any significant distinctions (*p* < 0.05).

## Result

### Pharmacokinetic result

**Fig. 1:**
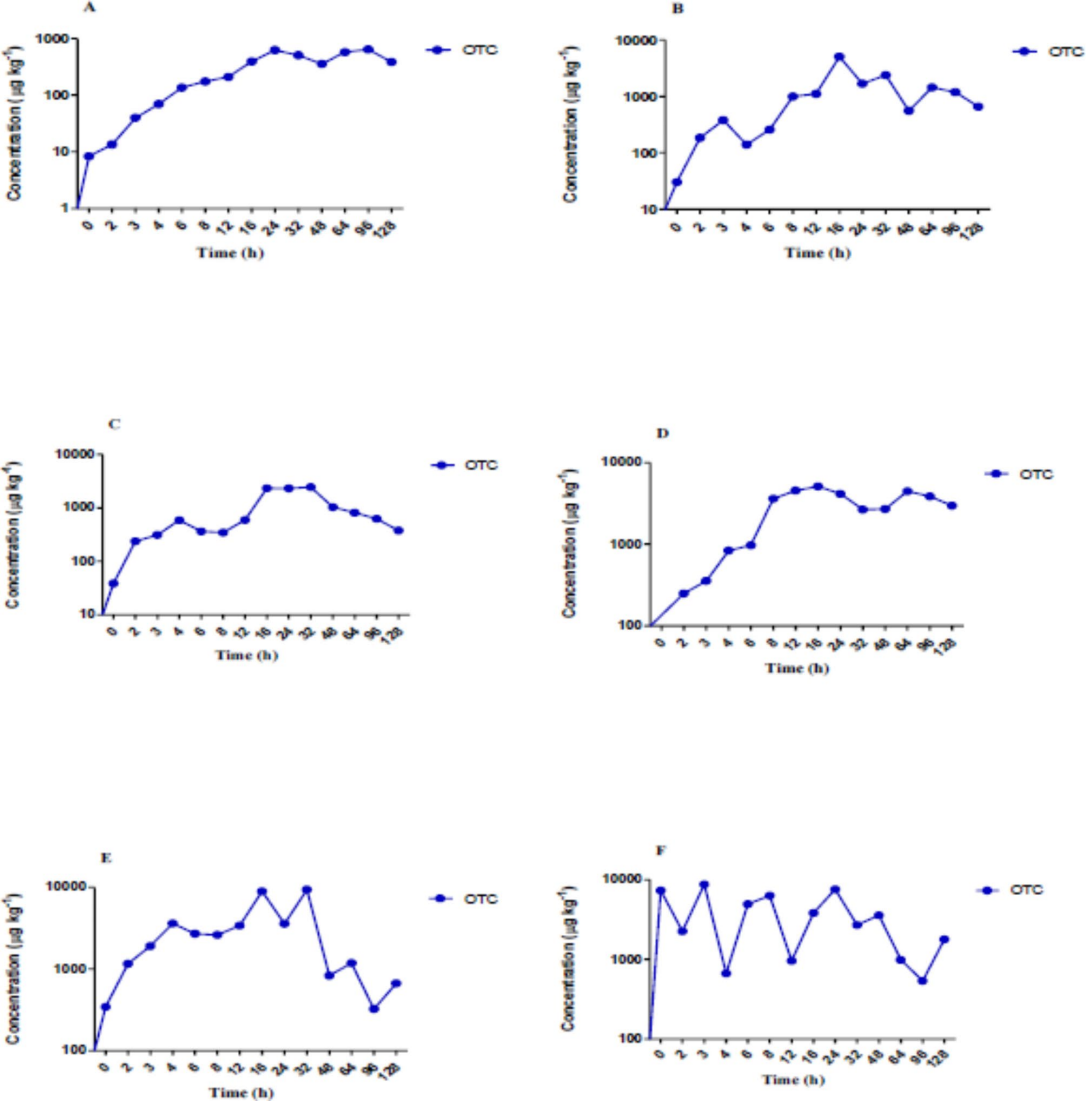
Antibiotic concentration vs. time curves of oxytetracycline dihydrate in rainbow trout after a single dose administration at 80 mg kg^−1^ fish body weight. (**A**) Plasma; (**B**) Skin; (**C**) Muscle; (**D**) Liver; (**E**) Kidney; (**F**) Gill.

The antibiotic concentration-time curves for the oxytetracycline dihydrate in plasma and other tissues are stated in Fig. 1(A–F). The pharmacokinetic characteristics of OTC were determined using a non-compartmental pharmacokinetic model based on statistical moment theory. The area under the concentration-time curve (AUC) was calculated using the trapezoidal method (Table 1). The data showed that oxytetracycline dihydrate was absorbed slowly with a very low absorption rate constant (*K*_*a*_) of 0.0002 to 0.011 h^−1^. The elimination rate constant (Λ) varied between tissues, with the highest value in the gill at 0.01 h^−1^. The half-life (t_1/2_) values varied among the tissues studied, with the shortest half-life observed in the gill tissue (0.94 h), followed by the liver (5.75 h), kidney (13.12 h), skin (9.88 h), and muscle (17.23 h). Interestingly, some tissues showed a distinction between the initial distribution phase (t_1/2a_) and the elimination phase (t_1/2b_). For example, in muscle tissue, the initial distribution half-life (t_1/2a_) was shorter (17.23 h) compared to the elimination half-life (t_1/2b_) (20.56 h), indicating a biphasic pattern. On the other hand, in the liver tissue, the elimination half-life (238.68 h) was substantially longer than the initial distribution half-life (5.75 h), suggesting a prolonged elimination phase. The duration of the elimination phase (T) also varied between tissues. The longest duration was observed in the liver at 606.33 h. The maximum concentration (*C*_*max*_) of the drug was in the kidney (9380 µg kg^−1^) and gill (8710 µg kg^−1^) and lowest in muscle (2460 µg kg^−1^). The time (*T*_*max*_) to reach *C*_*max*_ also varied between tissues, with the shortest time observed in the gill at 3 h and the longest in muscle at 32 h. The AUC_*(0−128)*_ values were highest in the liver and kidney at 450827.8 and 292651.3 µg h kg^−1^, respectively, and lowest in muscle at 129659.8 µg h kg^−1^. The AUC*(*_*tissue/plasma*_*)* ratio was highest in the liver and gill (7.34) and lowest in the muscle (2.81). The *k*_*el*_ values were negative for all tissues, indicating slow elimination of the drug. The AUC_*(0−∞)*_ values were negative in muscle and liver, indicating incomplete absorption and extensive distribution of the drug into these tissues.

**Table 1:** Pharmacokinetic parameters of oxytetracycline dihydrate post oral administration of medicated feed to rainbow trout at 80 mg kg^−1^ body weight.

S. no.	Pharmacokinetic parameter	Oxytetracycline					
		Plasma	Kidney	Gill	Liver	Muscle	Skin
1.	t_1/2a_ (h)	17.23	13.12	0.94	5.75	19.53	9.88
2.	t_1/2b_ (h)	137.36	14.30	55.67	238.68	20.56	36.03
3.	T	4859.34	227.68	90.79	606.33	273.18	286.34
4.	Λ	0.0002	0.0043	0.01	0.001	0.003	0.003
5.	*T*_*max*_(h)	24	32	3	16	32	16
6.	*C*_*max*_(µg kg^−1^)	627	9380	8710	5120	2460	5050
7.	AUC_*(0−128)*_	61414.6	292651.3	286732.3	450827.8	129659.8	173041.8
8.	AUC_*(tissue/plasma)*_		4.76	4.66	7.34	7.34	2.81
9.	*K*_*el*_	− 0.017	0.016	0.010	− 0.011	− 0.0007	− 0.0061
10.	AUC_*(t−∞)*_	− 22311.2	41425.63	182956.2	− 263,447	− 528,806	− 107,125
11.	AUC_*(0−∞)*_	39103.38	334076.9	469688.6	187380.4	− 399,146	65917.08
12.	K_a_ (h^−1^)	0.0002	0.0043	0.011	0.002	0.004	0.0034

*t*_*1/2a*_ half-life absorption, *t*_*1/2b*_ half-life elimination, *T* decay constant, *λ* mean life time, *T*_*max*_ maximum time, *C*_*max*_ maximum concentration, *AUC (h µg kg*^*−1*^*)* area under concentration-time curve, *K*_*el*_ elimination constant, *K*_*a*_ absorption rate constant.

### Absorption

During the absorption phase, the oxytetracycline dihydrate level in the kidney grew fast, peaking at 9380 µg kg^−1^ 32 h after the antibiotic was administered. The absorption rate reduced after 16 h, and the concentration declined to 324 µg kg^−1^ after 96 h. After 3 h, the gill had the highest concentration of oxytetracycline dihydrate (8710 µg kg^−1^). The liver and the skin contained concentrations of 5120 µg kg^−1^ and 5050 µg kg^−1^, respectively at 16 h, while the plasma contained a concentration of 627 µg kg^−1^ at 24 h, proportional to the fish body weight. The muscle had an oxytetracycline dihydrate concentration of 2460 µg kg^−1^ at 32 h post-treatment, which decreased to 376 µg kg^−1^ after 128 h.

### Distribution

The distribution phase of oxytetracycline dihydrate-mediated feed was observed in the plasma and gill immediately after oral administration, followed by the muscle, liver, and kidney at 2 h, and the skin at 3 h. This distribution phase lasted up to 16 h in all the tissues except in the plasma and gill, which persisted for 24 h, and the kidney, which lasted up to 32 h. The kidney exhibited the lowest concentration, reaching 2710 µg kg^−1^ at 8 h, while the muscle and liver reached concentrations of 362 µg kg^−1^ and 979 µg kg^−1^, respectively, at 6 h during the distribution phase. The skin and gill reached to lowest concentrations of 140 µg kg^−1^ and 666 µg kg^−1^, respectively at 4 h, while the plasma reached 13.50 µg kg^−1^ at 2 h. The concentration-time curve showed peaks at 6 h in the skin and gill, 3 h for the plasma, and 8 h for the gill. Moreover, in the drug-time curve, the primary peak for the muscle and kidney occurred at 12 h, indicating a priority for distribution during this stage. Comparing the area under the concentration-time curve (AUC) values, the relative distribution level of oxytetracycline dihydrate between the plasma and tissues after administration showed the highest AUC_(tissue/plasma)_ in the liver and muscle, with a value of 7.34 h µg kg^−1^, followed by the kidney (4.76 h µg kg^−1^), gill (4.66 h µg kg^−1^) and skin (2.81 h µg kg^−1^). These results indicated that OTC was widely distributed throughout all the examined tissues.

### Elimination

In the elimination phase, the kidney and muscle exhibited elimination of the antibiotic over a period of 32 h, while the gill took 48 h for the elimination. The elimination phase began post-64 h in the plasma, skin, and liver. During this phase, the drug levels declined rapidly to 386 µg kg^−1^ in plasma, 657 µg kg^−1^ in skin, 376 µg kg^−1^ in muscle, 2970 µg kg^−1^ in liver, 667 µg kg^−1^ in kidney, and 1790 µg kg^−1^ in gill. The elimination half-lives in plasma and other tissues, in decreasing order, were as follows; plasma (3368.24 h), liver (420.28 h), gill (198.48 h), kidney (189.35 h), skin (157.82 h), muscle (62.93 h), and gill (19.39 h).

### Tissue-specific validation parameters for oxytetracycline analysis

Table 2 shows the parameters used to validate the use of oxytetracycline in various tissues. The Limit of Detection (LOD) and Limit of Quantitation (LOQ) values varied significantly among the tissues tested, with some tissues showing negative values, indicating a minimal detectable concentration. For instance, the LOD values ranged from 44.88 µg kg^−1^ in plasma to -587.51 µg kg^−1^ in kidney tissue, while the LOQ values ranged from 136.00 µg kg^−1^ in plasma to -1780.34 µg kg^−1^ in kidney tissue. The R^2^ values, which reflect the linearity of the method, varied across tissues, with some tissues showing higher values than others. For example, muscle tissue had an R2 value of 6.96, while skin tissue had an R^2^ value of 0.002.

**Table 2:** Validation parameters for oxytetracycline.

S. no.	Parameter	Concentration (µg kg^−1^)					
		Plasma	Kidney	Gill	Liver	Muscle	Skin
1.	LOD	44.88	− 587.51	− 5.98	397.03	1123.08	116.98
2.	LOQ	136.00	− 1780.34	− 18.11	1203.12	3403.26	354.48
3.	R^2^	0.42	0.08	0.23	0.16	6.96	0.002

### Biosafety of oxytetracycline dihydrate

#### Behaviour and mortality

The behavior of fish in both the treatment and control groups was observed for seven days before commencing the biosafety experiment, which spanned over 40 days. During the experiment, some fish exhibited slow movement, lethargy, gasping for air, and light black pigmentation. Additionally, fish were observed near the tank inlet, displaying infections on their tails, and there were instances of fish mortality. Fish in all the treatment groups (80 to 800 mg kg^−1^) showed no mortality, demonstrating 100% survival.

#### Feed intake

The feeding behavior of trout appeared normal during acclimatization and the initial experimental phase. Throughout the study, the control group consistently exhibited typical feeding behavior. Conversely, each treatment group fish consumed 100% of the feed during the pre-medicated feeding period and post-medicated feeding periods. However, 80–90% of the feeding was recorded during the oxytetracycline-medicated feeding period (Table 3).

**Table 3:** Feeding behavior scoring on oral administration of oxytetracycline dihydrate in rainbow trout.

Periods	Feeding behavior scoring (mean ± SD)				
	Control	Oxytetracycline treated group 1 (mg kg^−1^ fish) 80	Oxytetracycline treated group 2 (mg kg^−1^ fish) 240	Oxytetracycline treated group 3 (mg kg^−1^ fish) 400	Oxytetracycline treated group 4 (mg kg^−1^ fish) 800
Pre medicated feeding period (0–10 days)	4 ± 0	4 ± 0	4 ± 0	4 ± 0	4 ± 0
OTC medicated feeding period (11–40 days)	4 ± 0	3.87 ± 0.34	3.7 ± 0.46	3.6 ± 0.60	3.5 ± 0.73
Post medicated feeding period (40–50 days)	4 ± 0	4 ± 0	4 ± 0	4 ± 0	4 ± 0

Based on the feed consumption, the feeding behavior of fish rated using a scale ranged from 0 to 4; (i) Scale 4 = 100% feed consumed, (ii) Scale 3 = 75% feed consumed, (iii) Scale 2 = 50% feed consumed, (iv) Scale 1 = 25% feed consumed, (v) Scale 0 = No feed consumed.

### Hematological parameter

**Fig. 2:**
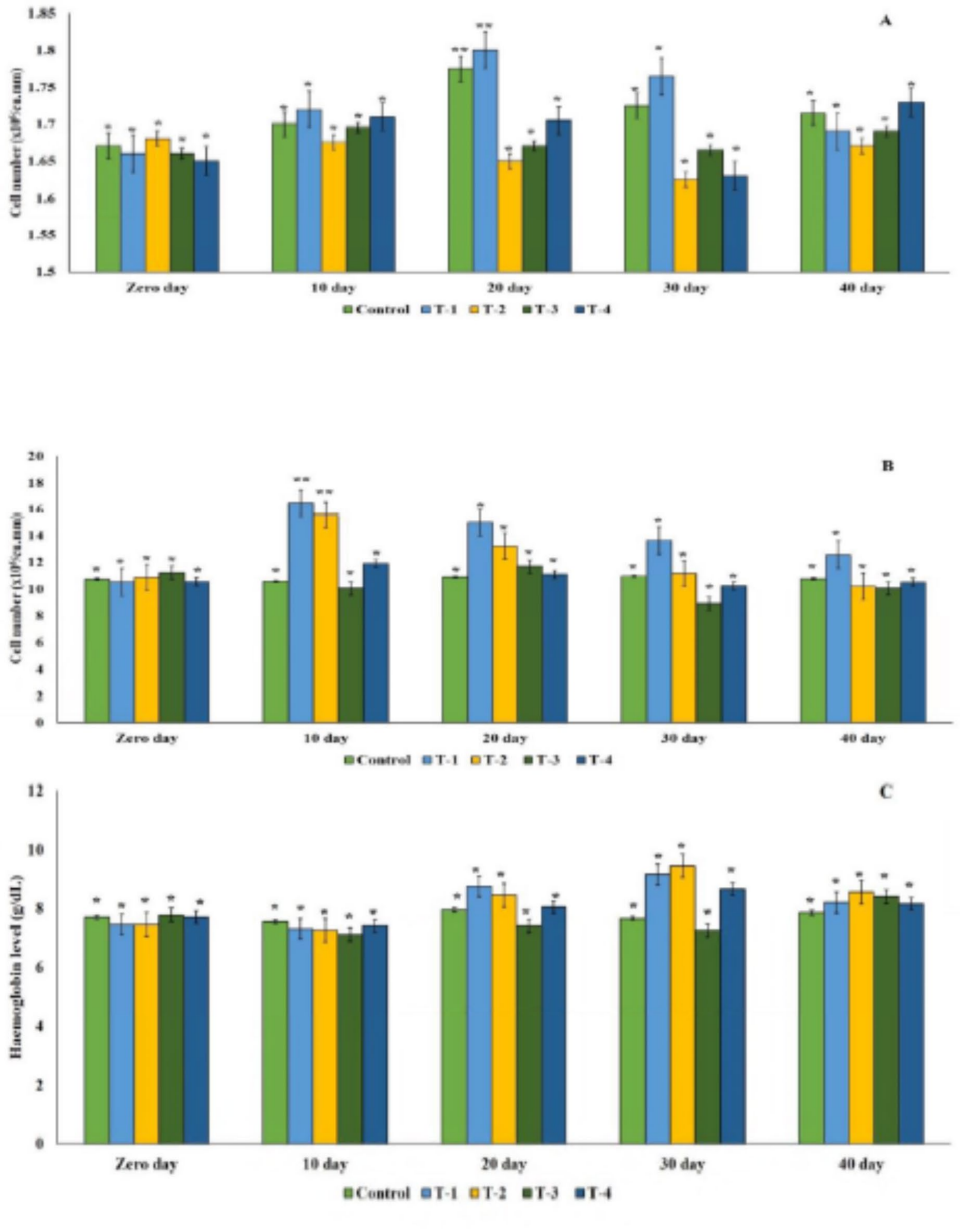
(**A–C**) Blood parameter profiles in control vs. oxytetracycline-treated Fish: (**A**) Erythrocyte Count, (**B**) Leucocyte count, (**C**) Haemoglobin Content. T-1: 80 mg kg^−1^ fish day^−1^; T-2: 240 mg kg^−1^ fish day^−1^, T-3: 400 mg kg^−1^ fish day^−1^ and T-4: 800 mg kg^−1^ fish day^−1^. ‘*’ and ‘*’: significance difference at *p* < 0.05.

In the biosafety study of oxytetracycline in rainbow trout, no significant difference in erythrocyte count was observed on the zero-day. However, in the 80 mg kg^−1^ treatment group, there was a gradual increase in erythrocyte count from 1.66 × 10^6^ per cu. mm on zero day to 1.8 × 10^6^ per cu. mm on day 20. The fish group treated with oxytetracycline dihydrate at 800 mg kg^−1^ showed a decline in erythrocyte count to 1.63 × 10^6^ per cu. mm on day 30. Leukocyte counts significantly increased in 80 mg kg^−1^ and 240 mg kg^−1^ groups after 10 days of antibiotic feeding and remained high up to 30 days. Hemoglobin levels insignificantly increased on day 30 in the 80 mg kg^−1^ and 240 mg kg^−1^ groups, measuring 9.15 g dl^−1^ and 9.44 g dl^−1^, respectively (Fig. 2A-C).

### Immune modulation

**Fig. 3:**
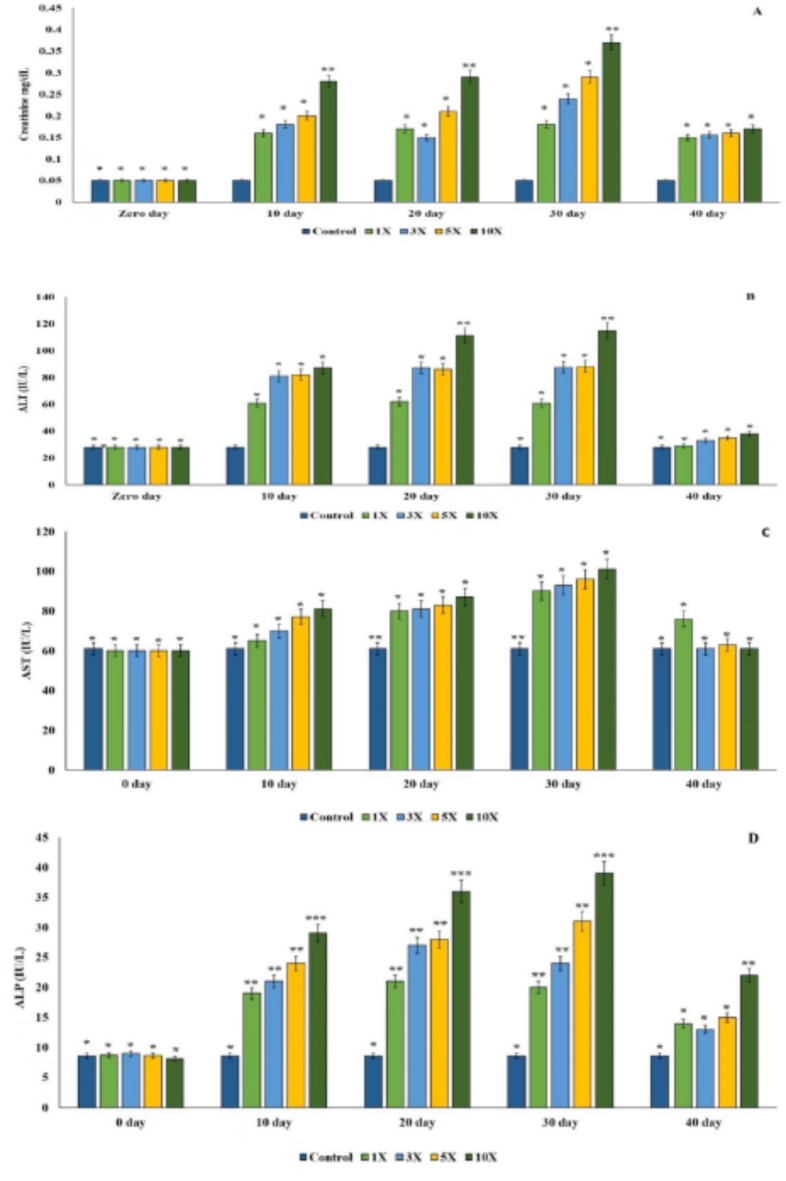
(**A–D**) Plasma immunomodulatory effects in rainbow trout administered oxytetracycline dihydrate at different multiples (1×, 3×, 5×, and 10×) of the therapeutic dose (80 mg kg^−1^ fish biomass day^−1^) for a continuous 30-day period. (**A**) Creatinine levels; (**B**) Alanine transaminase (ALT); (**C**) Aspartate transaminase (AST); (**D**) Alkaline phosphatase (ALP). ‘*’ and ‘*’: significance difference at *p* < 0.05.

In the control group, the serum creatinine level was observed to be 0.05 ± 0.001 mg dL^−1^. Following the administration of oxytetracycline dihydrate at various dosages in the treatment groups, there was a consistent increase in serum creatinine levels on the 30th day, which then began to decline after the antibiotic dosing was discontinued. Notably, no significant variation in creatinine levels was observed between the 1×, 3×, and 5× groups on the 10th day post-dosing of oxytetracycline dihydrate. However, it was evident that the creatinine level was significantly higher in the 10× concentration group compared to the other treatment groups (*p* < 0.05) (Fig. 3A). The Alanine transaminase (ALT) levels in the plasma of the control group were measured at 28.00 ± 1.06 IU L^−1^. ALT levels in the 1×, 3×, 5×, and 10× groups showed a notable increase from day 10 to the 30th day, followed by a decline after the 30th day. Importantly, no significant changes in ALT levels (*p* > 0.05) were observed in the control, 1×, 3×, 5×, and 10× groups after the 30th day of oxytetracycline dihydrate administration (Fig. 3B). There was a significant increase in Aspartate transaminase (AST) levels on the 20th and 30th day in the 1× group. Subsequently, the AST value gradually decreased from the 30th day onwards until the end of the experimental period. Similarly, the 3×, 5×, and 10× groups experienced an increase in AST levels until the 30th day of antibiotic dosing, after which their levels declined. Except for the 1× group, the AST levels in all other treatment groups returned to near-normal values on the 40th day (Fig. 3C). Throughout the observation period, the alkaline phosphatase (ALP) levels in the 1×, 3×, 5×, and 10× groups were consistently higher than those in the control group (*P* < 0.05). The ALP level in the 10× group was significantly the highest among all the groups (*p* < 0.05) and it declined 30th day (Fig. 3D).

### Histological alterations

**Fig. 4:**
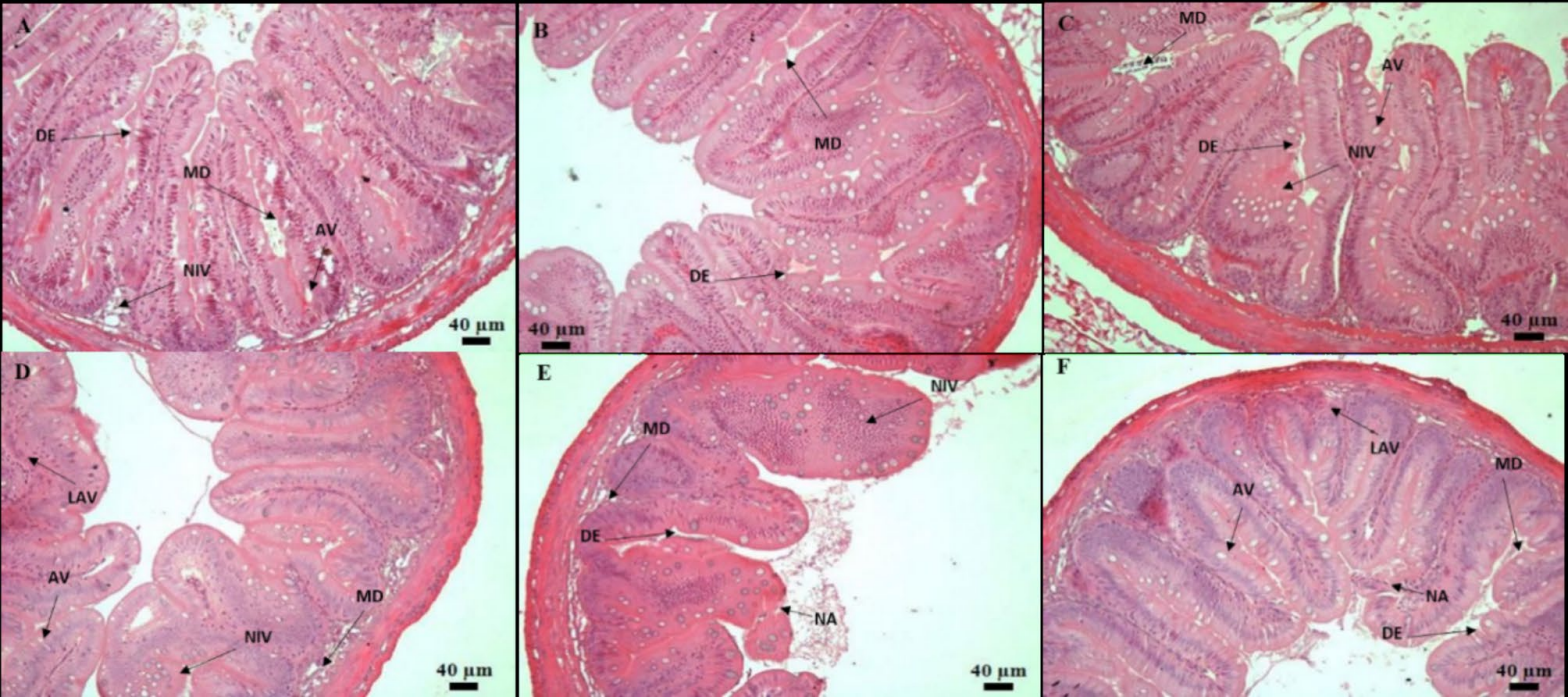
(**A–F**) Histological analysis of intestinal post exposure to antibiotic on 10th day—(**A**) 80 mg kg^−1^ fish day^−1^; (**B**) 400 mg kg^−1^ fish day^−1^; (**C**) 800 mg kg^−1^ fish day^−1^. Histological analysis of intestinal post exposure to antibiotic on 30th day—(**D**) 80 mg kg^−1^ fish day^−1^; (**E**) 400 mg kg^−1^ fish day^−1^; (**F**) 800 mg kg^−1^ fish day^−1^. Degenerated epithelial layer (DE), Intestinal villi necrosis (NIV), Mucinous degeneration (MD), Absorptive vacuole (AV), Necrotized absorptive region (NA), Loss of absorptive vacuole (LAV).

**Fig. 5:**
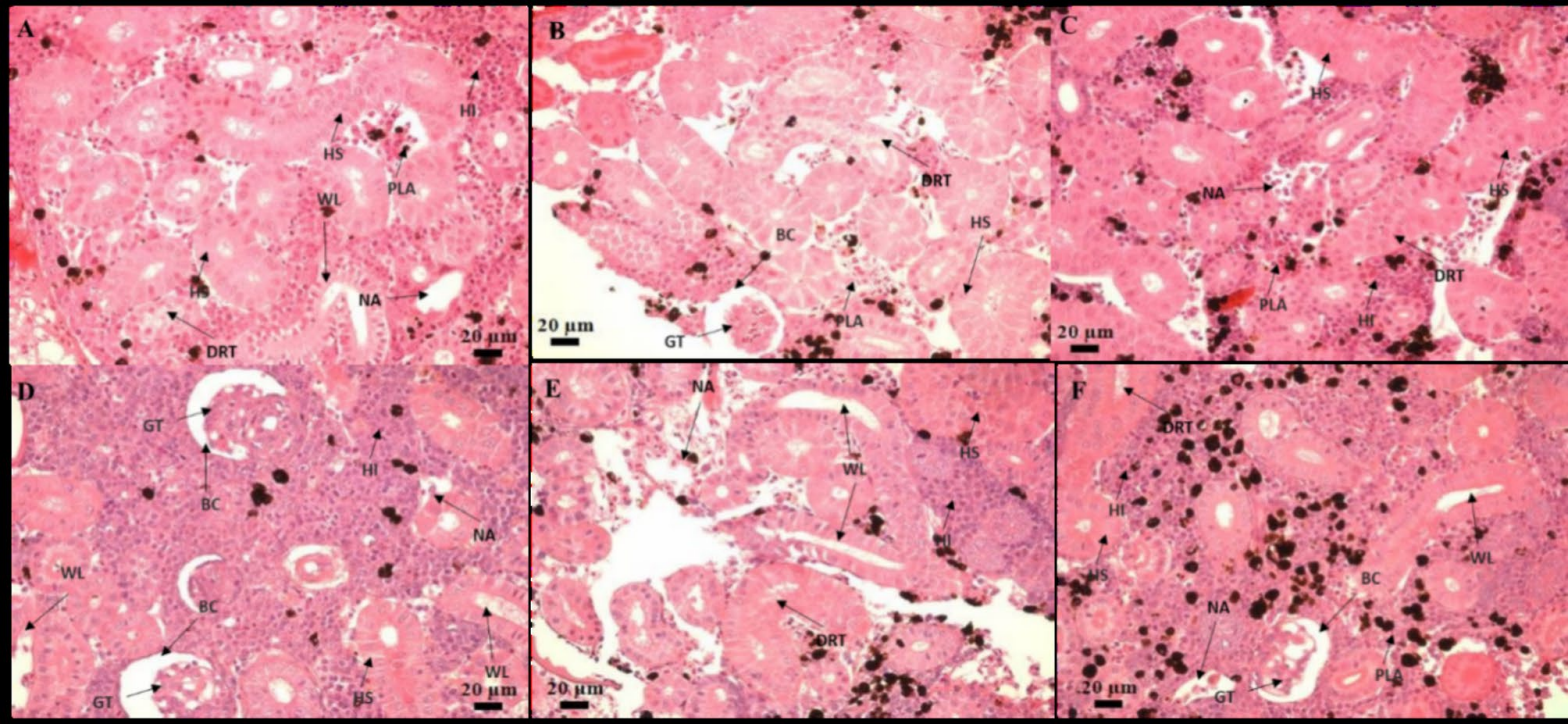
(**A–F**) Histological analysis of kidney post-exposure to antibiotic on 10th day—(**A**) 80 mg kg^−1^ fish day^−1^; (**B**) 400 mg kg^−1^ fish day^−1^; (**C**) 800 mg kg^−1^ fish day^−1^. Histological analysis of kidneypost-exposuree to antibiotic on 30th—(**D**) 80 mg kg^−1^ fish day^−1^; (**E**) 400 mg kg^−1^ fish day^−1^; (**F**) 800 mg kg^−1^ fish day^−1^. Histological changes included shrunken glomerular tufts (GT), Dilation of Bowman’s capsule (BC), Hydropic swelling (HS), Haemocyte infiltration (HI), Wide lumen (WL), Necrotized areas (NA), and Degeneration of renal tubule (DRT), along with Periglomerular lymphocytic aggregation (PLA).

**Fig. 6:**
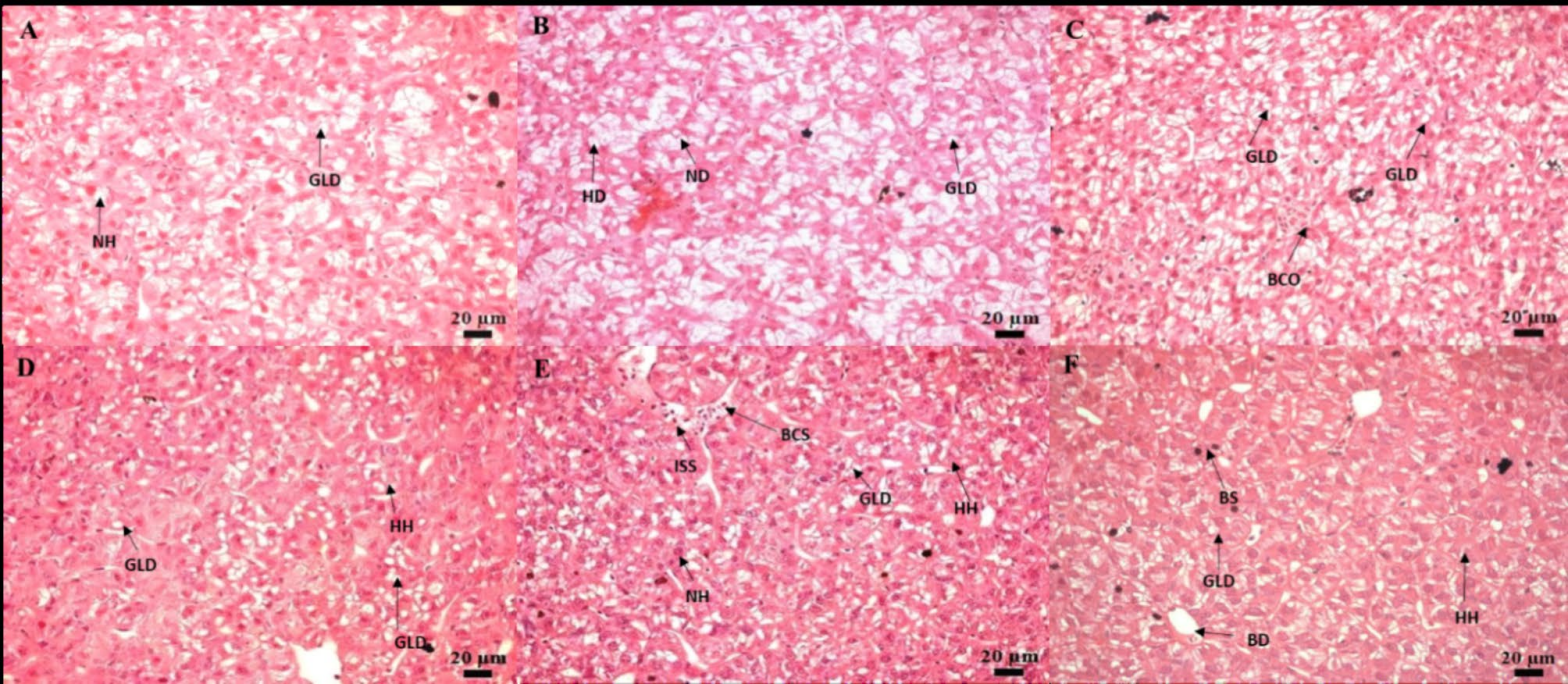
(**A–F**) Histological analysis of liver post exposure to antibiotic on 10th day—(**A**) 80 mg kg^−1^ fish day^−1^; (**B**) 400 mg kg^−1^ fish day^−1^; (**C**) 800 mg kg^−1^ fish day^−1^. Histological analysis of liverpost-exposuree to antibiotic on 30th day—(**D**) 80 mg kg^−1^ fish day^−1^; (**E**) 400 mg kg^−1^ fish day^−1^; (**F**) 800 mg kg^−1^ fish day^−1^. Histological changes included Hepatocytes (H), Hepatocyte Nucleus (HN), Glycogen/Lipid Droplets (GLD), Nuclear Hypertrophy (NH), Increase of Sinusoidal Space (ISS), Hepatocellular Degeneration (HD), Blood Congestion (BCO), and Nuclear Degeneration (ND) at 10 days. At 30 days, features encompass Hepatocytes (H), Hepatocyte Nucleus (HN), Blood Sinusoid (BS), Hepatocyte Hypertrophy (HH), Nuclear Hypertrophy (NH), Increase of Sinusoidal Space (ISS), Blood Congestion in Sinusoids (BCS), Glycogen/Lipid Droplets (GLD), Bile Duct (BD), and Bile Stagnation (BS).

**Fig. 7:**
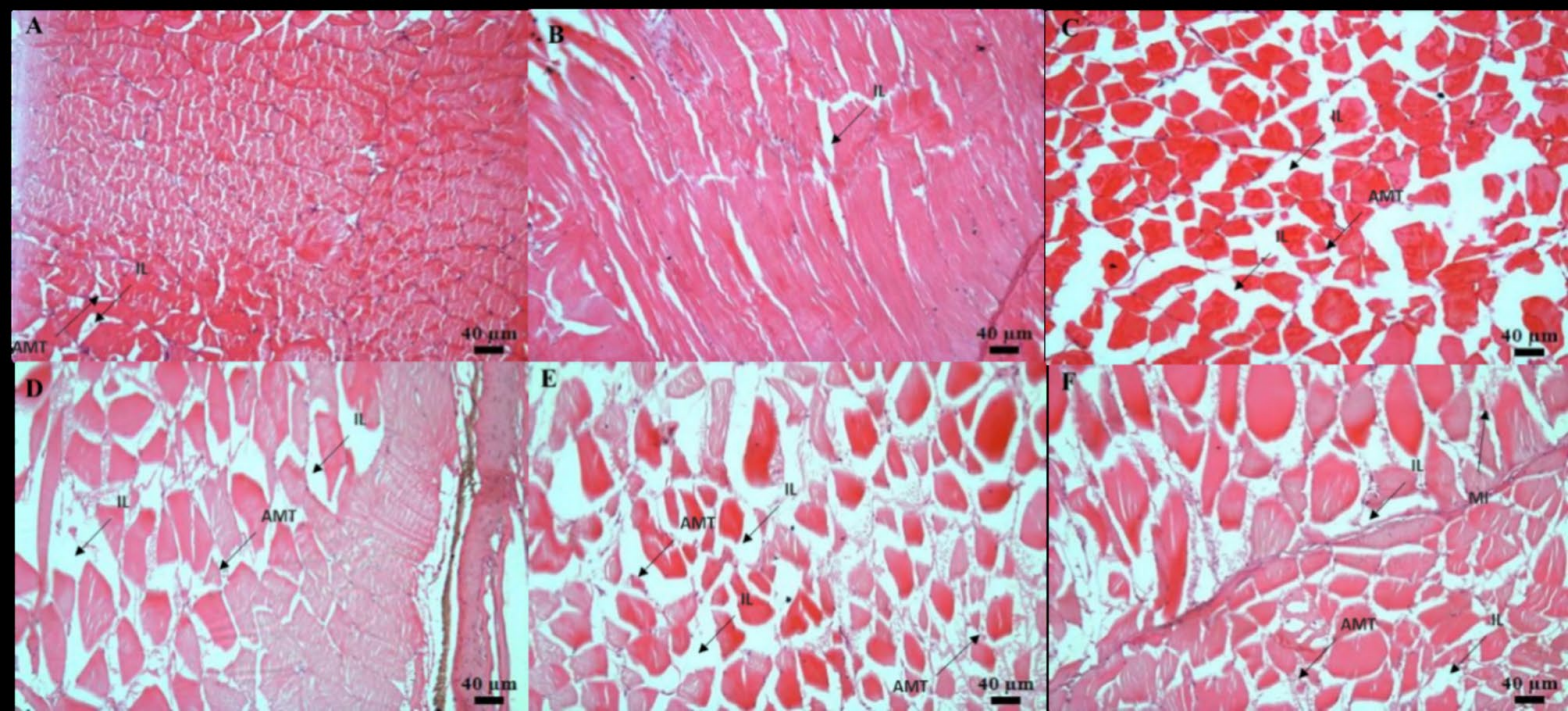
(**A–F**) Histological analysis of muscle post exposure to antibiotic on 10th—(**A**) 80 mg kg^−1^ fish day^−1^; (**B**) 400 mg kg^−1^ fish day^−1^; (**C**) 800 mg kg^−1^ fish day^−1^. Histological analysis of muscle post-exposure to antibiotic on 30th—(**D**) 80 mg kg^−1^ fish day^−1^; (**E**) 400 mg kg^−1^ fish day^−1^; (**F**) 800 mg kg^−1^ fish day^−1^. The histological changes included Interlobular Intervals (IL), Atrophy in Muscle Trabeculae (AMT), and Mild Inflammation (MI).

The histological analysis on the 10th and 30th days of sampling in the intestine revealed the presence of degenerated epithelial layers (DE), necrosis in intestinal villi (NIV), mucinous degeneration (MD), absorptive vacuoles (AV) necrotized absorptive regions (NA) and loss of absorptive vacuoles (LAV) at 1×, 5×, and 10× concentrations (Fig. 4A-F). The examination of the kidney demonstrated shrunken glomerular tufts (GT), dilation of Bowman’s capsule (BC), hydropic swelling (HS), hemocyte infiltration (HI), wide lumen (WL), necrotized areas (NA), degeneration of renal tubules (DRT), and periglomerular lymphocytic aggregation (PLA) in fish groups treated with OTC at 1×, 5×, and 10× concentrations (Fig. 5A-F). Further, as a consequence of prolonged exposure to oxytetracycline dihydrate at 1×, 5×, and 10× concentrations, the liver exhibited notable changes, including hepatocytes (H), hepatocyte nuclei (HN), blood sinusoids (BS), hepatocyte hypertrophy (HH), nuclear hypertrophy (NH), an increase in sinusoidal space (ISS), blood congestion in sinusoids (BCS), presence of glycogen/lipid droplets (GLD), bile ducts (BD) with bile stagnation (BS), blood congestion (BCO), and nuclear degeneration (ND) (Fig. 6A-F). The muscle displayed interlobular intervals (IL), atrophy in muscle trabeculae, and mild inflammation (MI) (Fig. 7A-F) (Table 4).

**Table 4:** Histological analysis of rainbow trout tissues post-antibiotic exposure at different dosages and time points.

Tissue	Day	Dosage	Histological analysis
Liver	10th	1×	Nuclear hypertrophy (NH), glycogen/lipid droplets (GLD)
		5×	Hepatocellular degeneration (HD), nuclear degeneration (ND), glycogen/lipid droplets (GLD)
		10×	Glycogen/lipid droplets (GLD), blood congestion (BCO)
	30th	1×	Glycogen/lipid droplets (GLD), hepatocyte hypertrophy (HH)
		5×	Increase of sinusoidal space (ISS), blood congestion in sinusoids (BCS), glycogen/lipid droplets (GLD), nuclear hypertrophy (NH), hepatocyte hypertrophy (HH)
		10×	Bile stagnation (BS), glycogen/lipid droplets (GLD), bile duct (BD), hepatocyte hypertrophy (HH)
Kidney	10th	1×	Hydropic swelling (HS); haemocyte infiltration (HI); wide lumen (WL); necrotized areas (NA) and degeneration of renal tubule (DRT); periglomerular lymphocytic aggregation (PLA).
		5×	Shrunken glomerular tufts (GT); dilation of Bowman’s capsule (BC), hydropic swelling (HS), degeneration of renal tubule (DRT); periglomerular lymphocytic aggregation (PLA).
		10×	Degeneration of renal tubule (DRT); periglomerular lymphocytic aggregation (PLA), hydropic swelling (HS), necrotized areas (NA), haemocyte infiltration (HI)
	30th	1×	Shrunken glomerular tufts (GT); Dilation of Bowman’s capsule (BC), hydropic swelling (HS); haemocyte infiltration (HI); wide lumen (WL); necrotized areas (NA)
		5×	Hydropic swelling (HS); haemocyte infiltration (HI); wide lumen (WL); necrotized areas (NA) and degeneration of renal tubule (DRT)
		10×	Shrunken glomerular tufts (GT); dilation of Bowman’s capsule (BC), hydropic swelling (HS); haemocyte infiltration (HI); wide lumen (WL); necrotized areas (NA) and degeneration of renal tubule (DRT); periglomerular lymphocytic aggregation (PLA).
Intestine	10th	1×	Degenerated epithelial layer (DE), necrosis in the intestinal villi (NIV), mucinous degeneration (MD), absorptive vacuole (AV)
		5×	Degenerated epithelial layer (DE), mucinous degeneration (MD)
		10×	Degenerated epithelial layer (DE), necrosis in the intestinal villi (NIV), mucinous degeneration (MD), absorptive vacuole (AV)
	30th	1×	Loss of absorptive vacuole (LAV), necrosis in the intestinal villi (NIV), mucinous degeneration (MD), absorptive vacuole (AV)
		5×	Degenerated epithelial layer (DE), necrosis in the intestinal villi (NIV), mucinous degeneration (MD), absorptive vacuole (AV)
		10×	Mucinous degeneration (MD), absorptive vacuole (AV), necrotised absorptive region (NA), loss of absorptive vacuole (LAV), degenerated epithelial layer (DE)
Muscle	10th	1×	Interlobular intervals (IL), atrophy in muscle trabeculae (AMT)
		5×	Interlobular intervals (IL)
		10×	Interlobular intervals (IL), Atrophy in muscle trabeculae (AMT)
	30th	1×	Interlobular intervals (IL), atrophy in muscle trabeculae (AMT)
		5×	Interlobular intervals (IL), atrophy in muscle trabeculae (AMT)
		10×	Interlobular intervals (IL), atrophy in muscle trabeculae (AMT), mild inflammation (MI)

### Analysis of antibiotic residues in muscle tissue

**Fig. 8:**
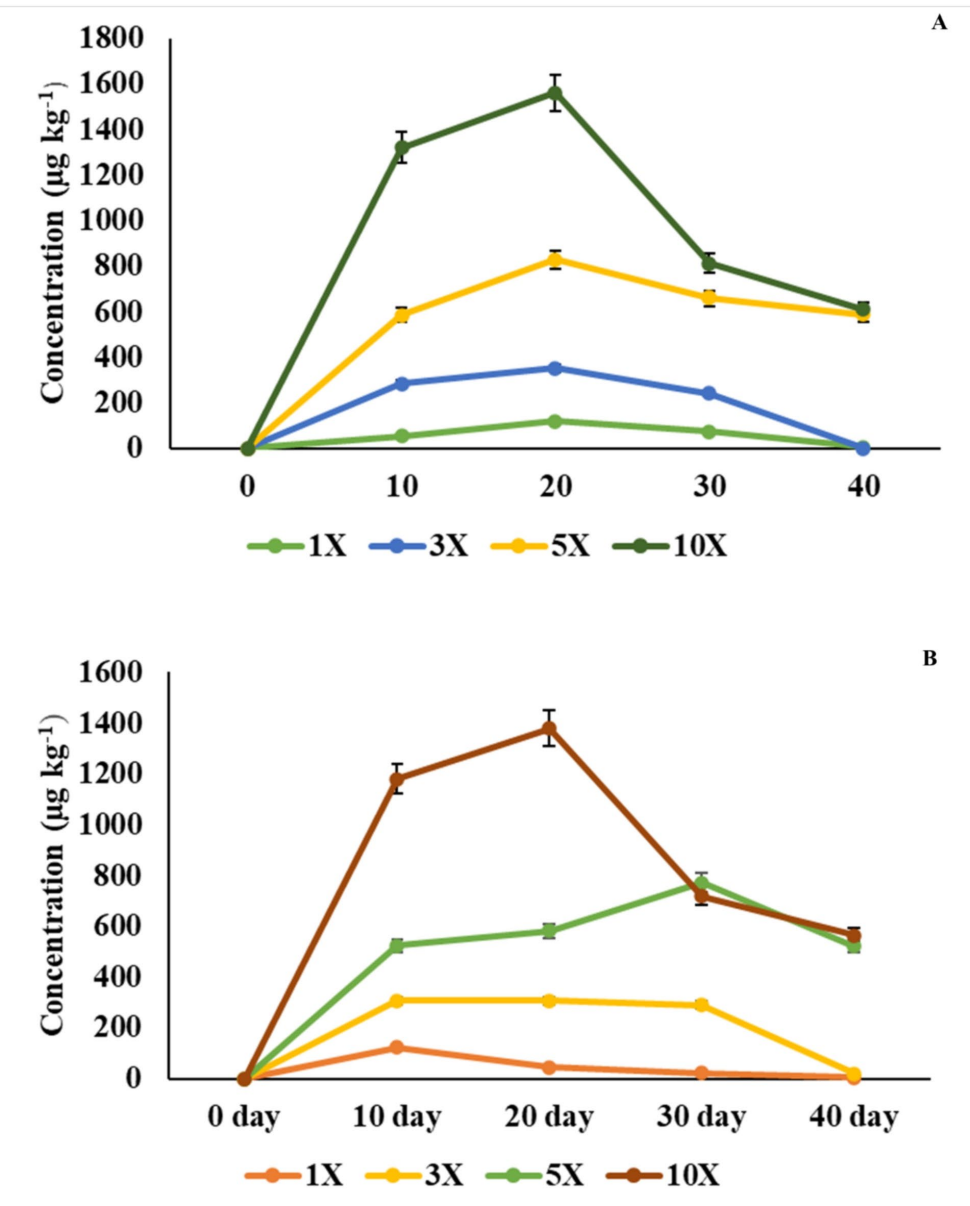
**(A**,** B)** Oxytetracycline residue levels in muscle tissue (µg kg^−1^) of rainbow trout, exposed to oxytetracycline and oxytetracycline epimer at 1×, 3×, 5×, and 10× concentrations of the therapeutic dose 80 mg kg^−1^ biomass day^−1^ over 30 days. (**A**) Oxytetracycline concentrations. (**B**) Concentration of oxytetracycline epimer.

Quantification of oxytetracycline dihydrate revealed varying prevalence of the drug at different time points, with the duration of dosage significantly correlating with muscle residue concentration (*p* < 0.05). On the 20th day, the maximum antibiotic concentration (*C*_*max*_) was 118, 351, 827, and 1560 µg kg^−1^ in the 1×, 3×, 5× and 10× groups, respectively. Subsequently, on the 40th day, the antibiotic residue concentrations in the muscle samples were decreased to 4.45, 1.29, 586, and 610 µg kg^−1^ in the 1×, 3×, 5×, and 10× groups, respectively (Fig. 8A).

**Fig. 9:**
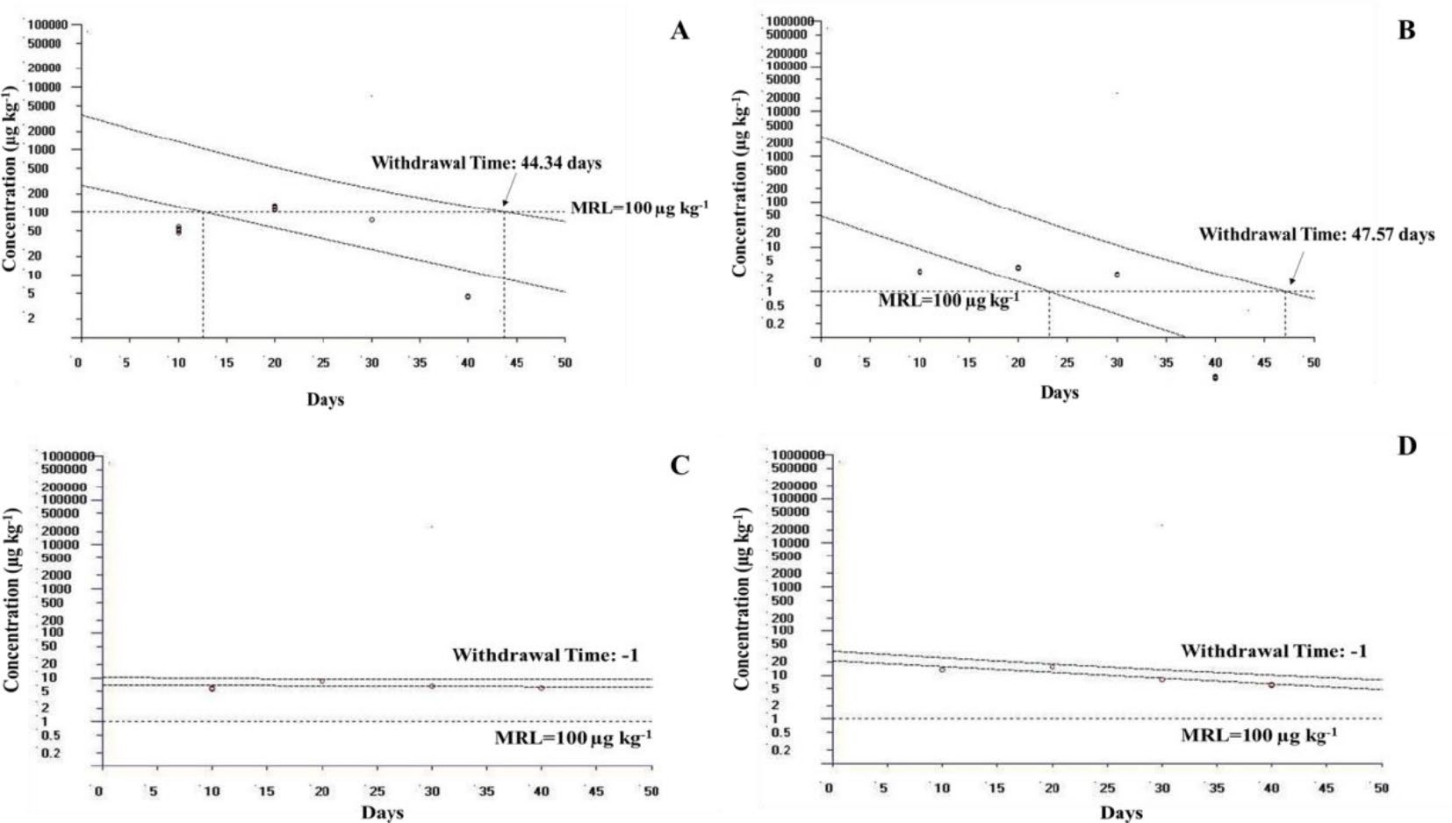
Estimated withdrawal times for oxytetracycline dihydrate in rainbow trout, following oral administration of OTC at 80, 240, 400 and 800 mg kg^−1^ fish bodyweight for 30 days. (**A**) The 1 × (80 mg kg^−1^) concentration shows a withdrawal time of 44.34 days. (**B**) The 3 × (240 mg kg^−1^) concentration shows a withdrawal time of 47.57 days. (**C**) The 5 × (400 mg kg^−1^) and (**D**) the 10 × (800 mg kg^−1^) concentrations indicate withdrawal time greater than 30 days (indicated by − 1). *MRL* maximum residue limits for Oxytetracycline from the European Medicines Agency (EMA).

The epimer of OTC, a *C*_*max*_ of 122 µg kg^−1^ was recorded at 1× concentrations on the 10th day (*T*_*max*_) of sampling, and it reached 772 µg kg^−1^ on the 30th day in 5×. At 3× and 10× concentrations, the residue concentrations were 308 and 1380 µg kg^−1^, respectively, on the 20th day. On the 40th day, the metabolite concentrations were 4.97, 18.9, 521, and 565 µg kg^−1^ at 1×, 3×, 5×, and 10× concentrations, respectively (Fig. 8B). Withdrawal times were 44.34 days for the 1× group and 47.57 days for the 3× group. In the 5× and 10× groups, the withdrawal time was more than 30 days (WT1.4 standard: -1) (Fig. 9).

### Water quality parameters

The average values of water quality parameters; pH (7.5), dissolved oxygen (7.8 mg L^−1^), alkalinity (80 mg L^−1^), water temperature (20.0 °C), calcium hardness (80 mg L^−1^), ammonia (0.02 mg L^−1^), nitrite (< 0.3 mg L^−1^), and nitrate (5 mg L^−1^) were within the acceptable range to avoid any stress factor for the experimental fish in both pharmacokinetics and biosafety trials. As experiment was conducted in controlled wet laboratory conditions, the water quality parameters were remained same for both the experiment.

## Discussion

The main objective of the study was to determine the pharmacokinetics parameter (PK) and biosafety for orally administered oxytetracycline dihydrate in rainbow trout. According to various studies^34–36^, oxytetracycline exhibits a low level of toxicity and has an excellent ability to disperse into the bloodstream and most tissues. However, its bioavailability, which refers to the total amount of the drug absorbed, is relatively limited due to its tendency to form complexes or chelate with polyvalent cations like Ca^++^, Fe^++^, Al^+++^, and Mg^++^^4^. These charged complexes, which are biologically inert, encounter difficulties in crossing lipid-rich biological membranes, leading to a significant decrease in the absorption of Oxytetracycline^4,37^. Moreover, oxytetracycline can also form complexes with organic material and clay. In fish, it is observed that oxytetracycline accumulates in bone tissue, the pronephros, and the scales of carp^38^. Fish do not significantly metabolize or bio-transform oxytetracycline, resulting in almost the entire administered dose being excreted or eliminated through defecation into the environment^37,39^. Specifically, it is estimated that approximately 60% of oxytetracycline is eliminated through urine via glomerular filtration, while the remaining 40% is excreted in feces^4^. Notably, the rate of oxytetracycline excretion is influenced by environmental temperature. A temperature-dependent effect on oxytetracycline excretion, with slower elimination observed at lower water temperatures^40,41^. Consequently, coldwater fish like trout and tench eliminate oxytetracycline at a slower rate compared to warm-water fish such as catfish, carp, and pacu, while cool-water fish like yellow perch likely exhibit an intermediate excretion rate^17^. Utilizing the characteristics related to how drugs are processed within the body (pharmacokinetics) and how they exert their effects (pharmacodynamics) presents a logical and effective strategy for enhancing the selection of suitable antimicrobial agents in clinical settings. However, the understanding of the impacts of various microbial agents, especially those that influence the functioning of vital organs responsible for drug processing, is rather limited in veterinary contexts, notably in aquatic animals^42^. Over the past decades, oxytetracycline has gained widespread usage in global finfish aquaculture. It has been applied for both therapeutic and preventive management of fish health^43,44^. The crucial aspect in establishing guidelines for drug withdrawal periods in rainbow trout was the identification of the maximum residual concentration that is considered safe for human consumption^41^. The investigation on withdrawal demonstrated a significant and uneven distribution of the drug within the body. Notably, the highest concentration of oxytetracycline was found in the intestine, followed by the liver, muscle, and kidney, and the lowest concentration was observed in the plasma. The distinctive accumulation of the drug in the liver and kidneys resulted in a reduction of effective blood concentration and concurrently increased the susceptibility of the organs to potential drug-related toxicity^45^. This phenomenon is believed to be a likely contributor to the development of hepatotoxicity and nephrotoxicity caused by oxytetracycline in various fish and animal species^8,11,12,46^. Both the kidneys and the digestive system, including the liver, play crucial roles in drug metabolism and excretion, leading to the observed elevated OTC concentrations in these organs. Nevertheless, it is important to note that the liver exhibited a notably longer duration for drug clearance compared to the kidneys. This is attributed to the hypothesis that continuous drug administration induces damage to the liver, resulting in an increase in drug accumulation and a decrease in clearance rate over time^47,48^. The study found that Oxytetracycline concentrations exceeded the Limit of Detection and Limit of Quantitation values. Negative LOD and LOQ values suggested minimal drug presence in samples, likely due to process variability, affirming method impartiality^49^.

The biosafety assessment of oxytetracycline was undertaken at 20 °C as drug metabolism is temperature-dependent^50^. During the oxytetracycline feeding period, a decrease in feed intake was noted in treatment groups, particularly with higher concentrations of antibiotic-treated feed, while the control group exhibited normal feeding behavior. This aligns with findings from previous studies suggesting reduced acceptability of medicated feed with increased drug doses^51–53^. Similar reductions in feed intake were reported in safety studies involving *P. hypophthalmus* and *Nile tilapia* exposed to elevated antibiotic dosages^47,54^. Oxytetracycline is known to suppress antibody production and reduce leukocyte levels^9^. The analysis of blood parameters revealed no significant or persistent changes in leucocyte numbers, except for a notable increase observed at the 1× concentration. Additionally, higher doses of the antibiotic led to an increase in hemoglobin levels and a decrease in erythrocyte counts, following the withdrawal of the drug, these deviations returned to baseline levels. The reduced erythrocyte counts and increased hemoglobin and leucocyte counts in *Labeo rohita* with prolonged exposure were observed^55^. The swift decline in drug concentrations post-withdrawal aligned with the restoration of various studied parameters to nearly normal values^47^.

Tetracyclines influence gene expressions associated with lipid metabolism in the liver, leading to the fat degeneration of hepatocytes^47^. Changes in liver enzymes ALT and AST serve as fundamental indicators of liver tissue damage due to stress, playing a crucial role in assessing liver metabolism^56^. The increase in ALT levels observed in the 1× group after 10 days of oxytetracycline dihydrate treatment compared to the control suggests potential liver tissue deterioration or indicates a mild injury to the liver^9^. The increase in ALT levels was noted in the 3×, 5×, and 10× groups from the 10th day to the 30th day, indicating hepatotoxicity^57^. An increased ALT level indicates moderate hepatic damage induced by oxytetracycline in treated fish^47^. It also induces genetic toxicity, leading to DNA damage, adduct formation, and DNA hypermethylation, which contributes to nephrotoxicity^9,11^. ALP is recognized for its role as an immune factor, offering protection against stress and infections^58^. The rise in ALP at the therapeutic dose suggested the potential for drug-induced liver inflammation and hepatotoxicity^59,60^. Until the 30th day of OTC treatment, ALP levels increased in a dose-dependent manner, with the highest values in the 10× group. Although ALP levels in all treatment groups decreased when oxytetracycline dihydrate medication was stopped, they remained considerably high compared to day zero, indicating persistent liver inflammation in the fish^13^. The rise in ALP levels is indicative of liver tissue necrosis^58^. The decrease in ALP levels after the 30th day signifies inhibited phosphorus metabolism in fish treated with antibiotics^47^. Large doses of oxytetracycline have deleterious effects on hepatorenal integrity in various animal and fish species^8,11,12^. Drugs or external stresses can cause increases in blood ALT and AST levels, indicating liver tissue injury^54,61^. The aspartate transaminase (AST) enzyme is abundant in the liver, heart, gill, kidneys, flesh, and other organs^13^. The increased levels of AST in the blood indicate the proper functioning of these organs^47^. On 10th day except for the 1× group, all other dosed groups showed an increase in AST levels confirming hepatotoxicity. Both amphenicols and oxytetracycline have been identified as hepatotoxic substances^54,62^. An increase in AST levels was also observed in *O. mykiss* after the administration of florfenicol^63^. AST and ALT, are classified as liver enzymes crucial for amino acid metabolism^64^. The increase in AST and ALT levels plays a significant role in determining damage levels in liver tissue and cell functions^65^. AST and ALT levels in the blood may indicate the breakdown of hepatocytes in liver tissue to meet the energy needs of fish due to liver shrinkage. Similar outcomes were reported in olive founder (*Paralichthys olivaceus*)^66^, Persian sturgeon (*Acipenser persicus*)^67^, and carp (*Cyprinus carpio*)^68^. All experimental groups showed an increase in creatinine levels after day 10, indicating kidney injury and a possible loss in renal function^54^. It is important to note that the pathological reaction can vary according to the fish species, dosage, and type of exposure^47^. The higher creatinine values suggest possible drug nephrotoxicity^50^. High levels of this clinical measure are frequently regarded as signs of organ damage, but an increase within normal or acceptable limits may also represent the adaptive reaction of the body to changes in bodily function^47^. Oxytetracycline demonstrated significant cytotoxic and genotoxic effects in the gill, liver, and kidney upon acute exposure to *Cyprinus carpio* in water^69^.

Histopathology serves as a validated ecotoxicological tool to analyze the outcomes of drug exposure in fish. It aids in detecting, defining, and quantifying pathological alterations occurring in specific key organs^70^. Histological changes, even with therapeutic doses, were evident from 10 days of exposure to Oxytetracycline, with the severity of pathological effects directly correlated with the dose and duration of the antibiotic treatment^46^. These histological changes in the kidney and liver indicated tissue impairment attributed to stress, toxicity, and liver damage during treatment. Notable features such as hepatocytes, blood sinusoid, hepatocyte hypertrophy, increase of sinusoidal space, and blood congestion were recorded in 5× and 10× concentration groups throughout the sampling period. Enzymatic activities like AST and ALT in blood substantiated these significant changes. Studies have reported histological changes in various fish species exposed to lower concentrations of oxytetracycline (100 to 150 mg kg^−1^ day^−1^), including *Tinca tinca*, *Coho salmon*, *O. niloticus*, *Gambusia holbrooki* and *Oncorhynchus mykiss*^70–74^. Similarly, investigations in the liver of *T. blochii* and *P. hypophthalmus* revealed characteristics such as diffuse congestion, hemorrhages, the presence of fatty cysts, and hyperplasia with increased vascularization of hepatocytes^46,47^. The liver, being the main target organ for antibiotic-induced damage, exhibited vacuolization attributed to excessive fat accumulation in the cytoplasm due to metabolic damage after exposure to various contaminants^75,76^. Hepatic vacuolization following exposure to tetracycline antibiotics has been reported in different fish species^70,71,74^. Higher doses of oxytetracycline (15 g kg^−1^ live weight) fed eight times induced dystrophic changes in the renal duct epithelium in *Cyprinus* carpio^77^, while periglomerular lymphocyte aggregation was noted in *O. niloticus* at lower doses of 100 mg kg^−1^ diet^9^. Mild degenerative changes in renal tubules, observed in groups receiving 240 and 400 mg kg^−1^, were reversed within ten days post-treatment termination^78^. These hepatotoxic effects are critical considerations, especially when fish are exposed to doses exceeding the recommended therapeutic levels or duration^46^. In the kidney, the enlargement of Bowman’s space indicates compromised glomerular filtration, resulting in inadequate removal of excess waste and fluids^58^. Muscle atrophy, characterized by reduced protein synthesis or loss, is often observed during prolonged skeletal muscle immobility. This condition involves diminished muscle contractile function and fiber size due to increased protein degradation and decreased synthesis^79–81^. Moreover, degenerative changes such as hyaline degeneration, atrophy, and inflammation were reported in muscle tissue^81^. In the intestine, various abnormalities including degenerated epithelial layer, necrosis in intestinal villi, mucinous degeneration, absorptive vacuole, necrotized absorptive region, and loss of absorptive vacuole were observed at different concentrations on the 30th and 40th day of sampling^13^.

Muscle tissue, the commonly consumed part of fish, was analyzed for oxytetracycline dihydrate residue using LC-MS/MS. Results indicated that tissue residual concentrations of the antibiotic remained below the maximum residue limits (MRL) set by the European Commission (100 µg kg^−1^)^82^ and the US Food and Drug Administration (200 µg kg^−1^)^83,84^ following prolonged therapeutic exposure for 30 days daily dosing of 1× and 3× concentrations. This led to withdrawal times of 44.34 and 47.57 days in 1× and 3×, respectively. In the 5× group, the antibiotic residue level reached 827 µg kg^−1^, while in the 10× group, it peaked at 1560 µg kg^−1^ by the 20th day after administration, leading to a withdrawal time exceeding 30 days. Subsequently, by the 40th day, the residue decreased to 586 µg kg^−1^ in the 5× group and 610 µg kg^−1^ in the 10× group, indicating the slow elimination of antibiotic residue from muscle tissue at 20 °C. Previous research demonstrated that oxytetracycline residue in muscle tissue and skin of *Nile tilapia* remained below the MRL 6-d post-treatment, subjected to a therapeutic exposure at 82.8 mg kg^−1^ body weight for 10 consecutive days^85^. Similarly, oxytetracycline residues in tilapia fillet were below the MRL (100 µg kg^−1^) 2 days post-treatment cessation, following exposure to the antibiotic at 80 mg kg^−1^ body weight for 5 days at water temperatures ranging from 16.5 to 24.5 °C^86^. The younger fish, with their higher metabolic rates, exhibited faster drug clearance^86^. The longer withdrawal time in the present study may be due to the prolonged therapeutic exposure (30 days) to daily dosing of OTC in rainbow trout at lower temperature of 20 °C. Overall, tissue residue concentrations in the muscle of fish exposed to oxytetracycline at therapeutic dosages for varying durations remained well below the MRL prescribed by regulatory bodies, ensuring consumer safety.

## Conclusion

The study investigating the pharmacokinetics and safety of oxytetracycline dihydrate administered through feed in rainbow trout, *O. mykiss* provided significant insights into distribution, absorption, and elimination of drug in different fish tissues. The pharmacokinetic analysis suggests that oxytetracycline is characterized by slow absorption and elimination in rainbow trout. Additionally, the drug demonstrates extensive distribution across various tissues, exhibiting diverse concentrations and elimination times. The liver and kidney have been identified as the primary organs responsible for metabolizing and eliminating the drug. The different half-lives in tissues, with the liver having the longest (238.68 h), indicated significant tissue retention and potential for prolonged therapeutic effects. Nevertheless, incomplete absorption observed in the muscle and liver highlights the necessity for further investigation to comprehensively comprehend the pharmacokinetics of oxytetracycline in these specific tissues. The findings strongly recommended utilization of oxytetracycline at a dosage of 80 mg kg^−1^ of fish body weight, which establishes a meticulous pharmacokinetic profile for oxytetracycline but delineates a robust biosafety foundation, positioning oxytetracycline as indispensable antibacterial agents in the dynamic realm of aquaculture.

## Data Availability

The data that support the findings of this study are included in the manuscript and are available from the corresponding author upon request.
